# Assessing the Link Between Statins and Insulin Intolerance: A Systematic Review

**DOI:** 10.7759/cureus.42029

**Published:** 2023-07-17

**Authors:** Karan Nareshbhai Dabhi, Namra V Gohil, Nida Tanveer, Sally Hussein, Shravya Pingili, Vijaya Krishna Makkena, Arturo P Jaramillo, Babatope L Awosusi, Javaria Ayyub, Tuheen Sankar Nath

**Affiliations:** 1 Internal Medicine, California Institute of Behavioral Neurosciences & Psychology, Fairfield, USA; 2 Internal Medicine, Medical College Baroda, Vadodara, IND; 3 Internal Medicine, Kakatiya Medical College, Hyderabad, IND; 4 Internal Medicine, Osmania Medical College, Hyderabad, IND; 5 General Practice, California Institute of Behavioral Neurosciences & Psychology, Fairfield, USA; 6 Pathology and Laboratory Medicine, California Institute of Behavioral Neurosciences & Psychology, Fairfield, USA; 7 Surgical Oncology, California Institute of Behavioral Neurosciences & Psychology, Fairfield, USA

**Keywords:** insulin intolerance, hmg-coa reductase inhibitors, lipid lowering agents, diabetes, statins, insulin resistance

## Abstract

There has been mixed and inconclusive evidence regarding the relationship between statin usage and insulin intolerance. This systematic review aims to comprehensively explore the link between the use of statins and insulin intolerance. We systematically searched MEDLINE, PubMed, PubMed Central (PMC), and Google Scholar databases for online English articles with full text. We excluded conference proceedings, editorials, commentaries, preclinical studies, abstracts, and preprints. The search across databases initially identified 667 articles. After eliminating duplicates and analyzing the remaining articles based on the inclusion and exclusion criteria, 11 articles were selected. The included studies had a total of 46,728,889 participants. The findings suggest that the use of statins is associated with a decrease in insulin sensitivity and insulin resistance. This systematic review provides evidence that the use of statins may have an adverse effect on insulin sensitivity and increase insulin resistance. These findings may have important clinical implications for individuals on statin therapy, especially those at risk of developing diabetes.

## Introduction and background

Statins, one of the safest medications used in clinical practice, are particularly effective in lowering cholesterol levels and are the most commonly given medication to treat hypercholesterolemia and the associated cardiovascular risks [1]. Statins can also lower postprandial hypertriglyceridemia, which lowers the risk of atherogenic plaque development [2]. As a result, statin medication is recommended for persons with a ten-year atherosclerotic cardiovascular disease risk of 20% or greater to lower low-density cholesterol (also known as LDL-c).

Despite their patients' high levels of atherosclerotic cardiovascular risk, certain healthcare providers are hesitant to recommend statins to pre-diabetic, dyslipidemic patients in light of recent findings [3]. The incidence of type 2 diabetes was considerably higher in the statin medication group by 12%, according to a recent meta-analysis of 29 randomized clinical trials (RCTs) [4]. However, given the homogeneous demographics and brief follow-up of clinical trials, use in real-world situations differs from use in those settings [5].

Uncertain mechanisms underlie the link between statin medication and diabetes [6]. Insulin resistance and increasing beta cell dysfunction contribute to the development of type 2 diabetes, with the latter being necessary for the transition to overt diabetes. There is disagreement on the effects of statin therapy on insulin sensitivity, and studies are typically small in size [7-10]. Simvastatin and rosuvastatin treatments have reduced insulin sensitivity, whereas pravastatin treatments have increased it [11,12]. These studies have sparked a discussion regarding how statins' potential to increase diabetes risk should be weighed against the projected cardiovascular risk advantages of lowering LDL cholesterol. Some in vitro investigations, but not all, have observed a statin-induced reduction in insulin secretion [13].

As the relationship between the use of statins and incident diabetes is still unclear, statins have other health advantages besides just lowering cholesterol, such as reducing systemic inflammation and oxidative stress and improving endothelial function, all of which would improve rather than hinder carbohydrate metabolism [14]. Furthermore, dyslipidemia, a condition with abundant circulating fat, would encourage the buildup of non-esterified intermediates from lipid metabolism in tissues, which would block insulin signaling [15]. Statins should therefore prevent rather than exacerbate insulin resistance because they lower dyslipidemia (LDL cholesterol and triglycerides). In this study, we intended to shed more light on the diabetogenic effects of statins through a thorough examination and synthesis of the literature published during the last 10 years.

There has been mixed and inconclusive evidence during the past few years regarding the relationship between statin usage and insulin intolerance/ insensitivity. The study aimed to assess the link between the use of statins and insulin intolerance.

## Review

Materials and methods

In conducting this systematic review and presenting the findings, we followed the Preferred Reporting Items for Systematic Reviews and Meta-Analyses (PRISMA) guidelines and principles as outlined in the reference [16].

Search Strategy

To find relevant articles, we searched major research literature databases and search engines like MEDLINE, PubMed, PubMed Central (PMC), and Google Scholar using appropriate keywords and the Medical Subject Headings (MeSH) thesaurus on the 9th of April 2023 [17-19].

Here is the MeSH strategy that we used for PubMed, PMC, and MEDLINE: "Hydroxymethylglutaryl-CoA Reductase Inhibitors"[MeSh] AND "Insulin Resistance"[MeSh].

The keywords used for search in Google Scholar include "Statins", "Insulin resistance", "Diabetes", "Lipid-lowering agents", "Insulin Intolerance", "Hydroxymethylglutaryl-CoA Reductase Inhibitors", and "HMG-CoA Reductase Inhibitors". To discover pertinent articles, we utilized Boolean operators such as "AND", "OR", and "NOT" to combine different keyword combinations.

Inclusion and Exclusion Criteria

We thoroughly researched observational studies, randomized controlled trials (RCTs), reviews, and meta-analyses published in English within the past decade. Our aim was to focus on individuals aged 18 years and older, including geriatric patients. We did not consider articles related to the pediatric population, case reports, letters, expert opinions, animal studies, or unpublished and non-peer-reviewed literature. Inclusion and exclusion criteria are given in Table 1.

**Table 1:** Detailed inclusion and exclusion criteria

Inclusion criteria	Exclusion criteria
Studies that investigate the relationship between statin use and insulin intolerance or resistance.	Studies that do not investigate the relationship between statin use and insulin intolerance or resistance.
Studies that include human participants.	Studies that do not include human participants.
Studies that report on outcomes related to insulin intolerance or glucose metabolism, such as insulin resistance, fasting blood glucose, hemoglobin A1c, or oral glucose tolerance test results.	Studies that do not report outcomes related to insulin intolerance or glucose metabolism.
Studies that are published in English.	Studies that are not published in English.
Studies that have been published in the past 10 years.	Studies that have been published in the past 10 years.
Systematic reviews, meta-analyses, randomized control trials, and other observational studies.	Case reports, letters, expert opinions, animal studies, grey literature, and unpublished literature.
Papers focused on adults and the elderly population over 18 years old.	Papers discussing individuals under 18 years of age.

Quality Assessment

We conducted a thorough evaluation of 12 studies using standardized quality assessment tools. Of the 12 studies, 11 were considered of good or fair quality and included in the review. We used the Newcastle-Ottawa scale (NOS) to assess the quality of observational studies. For systematic reviews and meta-analyses, we employed the Assessment of Multiple Systematic Reviews (AMSTAR) tool, and for RCTs, we used the Cochrane risk-of-bias assessment tool.

Data Extraction

The data was gathered from qualified studies by two reviewers, including the lead author and publication year, study design and settings, the number of participants, the outcomes related to insulin intolerance (such as changes in insulin sensitivity, glucose metabolism, and glycemic control), and the findings from the studies. The data extraction process was carried out using a standardized form, and any discrepancies were resolved through discussion and consensus. The collected data was thoroughly analyzed and combined to offer a complete understanding of the link between insulin intolerance and statin use. Any possible sources of bias or differences were also identified during the process.

Results

Study Selection

**Figure 1:**
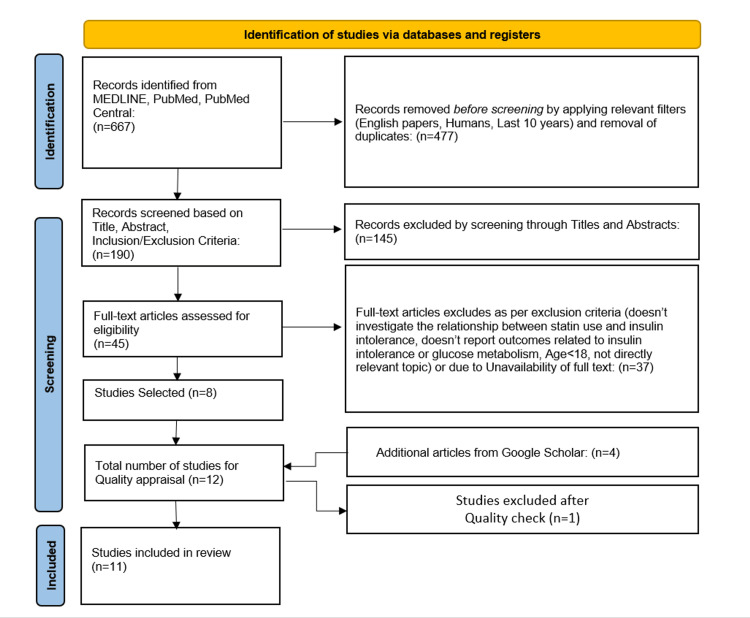
Preferred Reporting Items for Systematic Reviews and Meta-Analyses (PRISMA) flowchart

The literature search from PubMed, PMC, and MEDLINE yielded 667 articles. After applying the ten-year filter and removing duplicates, 477 articles were eliminated. One hundred and forty-five articles were excluded while screening titles and abstracts for being irrelevant to the research question. The remaining 45 articles were read in total, and 37 articles were excluded at this stage. The 37 articles excluded were of the study methodology stated in the exclusion criteria, did not report the link between statin usage and insulin intolerance, or lacked full text. The remaining eight articles and four other articles identified from Google Scholar were submitted for quality assessment, and one study was eliminated. Finally, only 11 studies ultimately agreed with the inclusion criteria and had acceptable quality. The selected studies had a combined total of 46,72,889 participants. A flowchart depicting the process of study selection is presented in Figure 1 [16].

Characteristics of the Included Studies

The years of publication range from 2013 to June 2022; three of them were RCTs [8,20,21], six were cohort studies [9,10,22-25], and two were systematic reviews and meta-analyses [5,26]. Most studies were conducted in Europe (five) and the US (four). Study characteristics and findings are summarized in Table 2.

**Table 2:** Traits of the studies that have been incorporated RCT = randomized controlled trial; LDL-c = lower low-density cholesterol; NA = not applicable; USA = United States of America

Author and Publication year	Study settings	Study design	Sample size	Patient’s age	Gender (male/female)	Outcome measured	Findings from the study
Olotu et al. (2016) [24]	USA	Cohort	106,424	20–63 years	54398/52026	Incident diabetes	Statin treatment is significantly linked with an increased risk of type 2 i diabetes.
Koh et al. (2016) [20]	South Korea	RCT	190	Mean age 57 years	95/95	Glycated hemoglobin levels, adiponectin levels, and insulin resistance	Rosuvastatin substantially and dose-dependently decreased insulin sensitivity and raised ambient glycemia.
Ko et al. (2019) [23]	South Korea	Cohort	1034982	aged ≥40 years	397407/637575	Incident diabetes	The risk of newly developing diabetes mellitus increased with statin use in a time- and dose-dependent manner.
Yoon et al. (2016) [25]	South Korea	Cohort	41325	Mean age 54.2 years	20950/20375	Incident diabetes	In a real healthcare context, statin users were found to have a higher chance of developing new-onset diabetes mellitus in Korea.
Carter et al. (2013) [12]	Canada	Cohort	471250	66 years or older	216304/254946	Incident diabetes	Treatment with more potent statins, particularly atorvastatin and simvastatin, may raise the chance of developing new cases of diabetes.
Puurunen et al. (2013) [21]	Finland	RCT	38	29 –50 years	0/38	Insulin sensitivity, fasting glucose, and fasting insulin levels	Treatment with atorvastatin decreases insulin sensitivity in women while improving lipid profiles and chronic inflammation.
Cederberg et al. (2015) [10]	Finland	Cohort	8,749	45–73 years	8749/0	Insulin sensitivity, fasting glucose, incident diabetes and insulin secretion	Due to reductions in insulin sensitivity and secretion, statin therapy elevated the incidence of type 2 diabetes by 46%.
Alvarez‐Jimenez et al. (2022) [8]	Spain	RCT	21	mean age 61 ±7 years	20/1	Insulin sensitivity and fasting glucose	Withdrawal of statin therapy had little effect on pre-diabetic hypercholesterolemic individuals' fasting or post-meal insulin resistance.
Ahmadizar et al. (2019) [9]	Netherlands	Cohort	9535	45 years or older	3976/5559	Fasting insulin levels, insulin sensitivity and incident diabetes	Statin users may be more likely to develop hyperglycemia, insulin resistance, and, finally, type 2 diabetes.
Casula et al. (2017) [5]	Italy	Meta-analysis	3020446	NA	NA	Incident diabetes	The study confirmed and reinforced the evidence that statins have diabetogenic properties.
Cai et al. (2014) [26]	China	Meta-analysis	95 102	NA	NA	Incident diabetes	Statin medication with a lower enhanced target LDL-c level increased the likelihood of developing diabetes.

Cohort Studies Quality Assessment Results

The observational cohort studies were assessed using the NOS: All six studies truly represented the patients included. Likewise, the control group was selected from the same community. Secure records confirmed the ascertainment of exposure. Also, the two groups included in all studies were comparable. They all also showed adequate follow-up. Therefore, the overall quality of all the cohort studies is good (Tables 3, 4).

**Table 3:** A brief overview of the Newcastle-Ottawa risk of bias for cohort studies OOI = outcome of interest. Quality score ratings all fall within the “good” range

	Cohort studies								
ID	Selection					Outcome			
	Representativeness	Selection	Ascertainment	OOI absent at study onset	Comparability	Assessment	Follow-up period duration	Follow-up quality	Quality score
Ahmadizar et al. (2019) [9]	1	1	1	1	1	1	1	1	8
Olotu et al. (2016) [24]	1	1	1	1	1	0	1	1	7
Cederberg et al. (2015) [10]	1	1	1	1	2	1	1	1	9
Yoon et al. (2016) [25]	1	1	1	1	2	1	1	1	9
Carter et al. (2013) [12]	1	1	1	1	2	1	1	1	9
Ko et al. (2019) [23]	1	1	1	1	2	1	1	1	9

**Table 4:** A brief overview of the Newcastle-Ottawa risk of bias for cross-sectional study Quality score ratings fall within the “poor” range

Cross-sectional studies								
ID	Selection					Outcome		
	Representativeness	Sample size	Ascertainment	Non-respondents	Comparability	Assessment	Reporting the results	Quality score
Thomson 2018	1	0	1	0	0	1	1	4

Randomized Controlled Trials (RCTs) Quality Assessment Results

**Figure 2:**
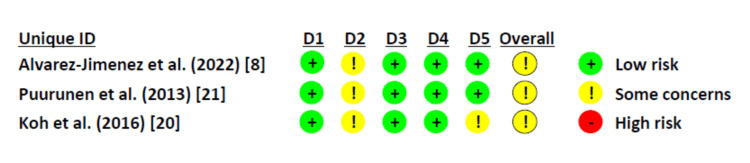
Risk of bias summary for randomized controlled trials D1: Randomization process; D2: Deviations from the intended interventions; D3: Missing outcome data; D4: Measurement of the outcome; D5: Selection of the reported result

**Figure 3:**
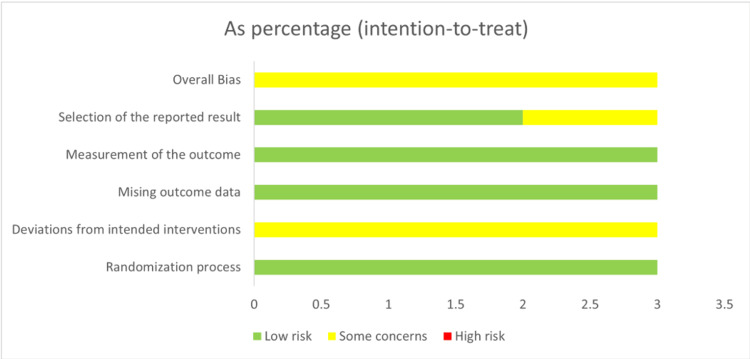
Risk of bias graph for randomized controlled trials

RCTs were assessed using the Risk of Bias 2 (Rob2) tool as follows: All three included trials reported the correct method of random sequence generation through computer-generated random sequences. Allocation concealment was achieved in the three studies through sealed envelopes. In addition, all the studies had no baseline differences between the intervention groups. Double-blinding was not achievable in the two studies, but this did not substantially impact the results. Koh 2016 [20] achieved only single blinding. None of the three studies reported an appropriate analysis method; therefore, they were all judged to have some concerns. All the studies reported nearly complete outcome data. All the studies showed correct outcome measurements. However, blinding of the outcome assessors was reported in two studies (Jimenez, 2022; Koh, 2016) [8,20]. None of the three studies reported results according to a registered protocol or pre-specified analysis plan. The risk of bias summary and graph are shown in Figures 2, 3.

Meta-Analysis Quality Assessment Results

The quality appraisal of two meta-analysis studies included in this systematic review was done using the AMSTAR tool, and their results are shown in Table 5.

**Table 5:** Meta-analysis quality assessment results AMSTAR = Assessment of Multiple Systematic Reviews; PICO = patient/population, intervention, comparison, outcome; RoB = risk of bias

AMSTAR criteria	Study 1	Study 2
	Cai et al. (2014) [26]	Casula et al. (2017) [5]
Were the PICO components included in the research questions and inclusion criteria for the review?	Yes	Yes
Was there a clear declaration in the review report that the review methods were established before the review was conducted? Additionally, did the report provide reasoning for any major deviations from the protocol?	No	No
Were the criteria for selecting the study designs for the review explained by the authors?	No	Yes
Was a thorough literature search strategy utilized by the authors of the review?	Yes	Yes
Did the authors of the review conduct a duplicate study selection?	Yes	Yes
Were the review authors able to perform duplicate data extraction?	Yes	Yes
Did the authors of the review give a list of studies that were not included and explain why they were excluded?	Yes	Yes
Did the authors of the review provide sufficient details about the studies included?	Yes	Yes
Did the authors of the review use a reliable method to evaluate the RoB in the studies that were included in the review?	Yes	Yes
Did the authors of the review disclose the funding sources for the studies that were part of the review?	No	No
Did the review authors use appropriate methods for statistical combination of results if they performed a meta-analysis?	Yes	Yes
Did the review authors consider the potential impact of RoB in individual studies on the results of the meta-analysis or other evidence synthesis, if they performed a meta-analysis?	Yes	Yes
When interpreting and discussing the results of the review, did the authors consider the risk of bias in each individual study?	Yes	Yes
Did the authors of the review adequately explain and discuss any differences observed in the review results?	Yes	Yes
Did the review authors perform a sufficient investigation of publication bias (a slight study bias) and discuss its potential impact on the review results if they conducted quantitative synthesis?	Yes	Yes
Did the authors of the review disclose any potential conflicts of interest, such as funding they may have received for the review?	Yes	No
Total score	13/16 (high quality)	13/16 (high quality)

Discussion

Unknown mechanisms underlie the link between statin medication and insulin sensitivity [6]. This study is a systematic review aiming to investigate the link between statins and insulin intolerance/insensitivity. A total of 11 epidemiological studies assessing the link between statins and insulin sensitivity met our inclusion criteria.

Effect of Statins on Fasting Glucose, Hemoglobin A1c, and Fasting Insulin

Fasting insulin, fasting glucose, and hemoglobin A1c (HbA1c) are all biomarkers frequently employed to evaluate insulin sensitivity and keep track of blood sugar levels. Fasting insulin measures the blood's insulin level after fasting for at least eight hours. In contrast, fasting glucose measures the blood's glucose level after an individual has fasted for at least eight hours [27]. Lowered insulin sensitivity and insulin resistance are signs that the body is not using insulin as efficiently as it should, which might be indicated by high fasting glucose (hyperglycemia) or high fasting insulin levels (hyperinsulinemia) [27]. The HbA1c test measures the average blood glucose level over the past three months. It displays the proportion of glucose-bound hemoglobin molecules. A higher HbA1c indicates poorer glucose control, linked to lower insulin sensitivity and an increased risk of consequences such as retinopathy, nephropathy, and neuropathy [28].

Five studies reported the effect of statins on fasting glucose, HbA1c, and fasting insulin concentrations [8-10,20,21]. Evidence from four of the five studies shows that statin usage increases the concentrations of fasting glucose, HbA1c, and fasting insulin. However, one study by Alvarez-Jimenez et al. discovered that statin withdrawal has no impact on fasting insulin or glucose levels, indicating that in hypercholesterolemic people, chronic statin treatment does not worsen pre-diabetes (i.e., insulin resistance) [8]. Two main criticisms of the study by Alvarez-Jimenez et al. may explain the conflicting results. First, the lack of change in glucose or insulin responses could be because 96 hours of drug removal is insufficient time to see the effects of statins causing insulin resistance. Second, the study found that statins have long-lasting and potentially irreversible effects on diabetes, which were only discovered in individuals who had taken the medication for over three years [8].

Regarding dosage, statin affects fasting glucose concentrations, and fasting insulin is dose-dependent [20]. Higher doses of statins (20 mg) are associated with high concentrations of fasting glucose and fasting insulin compared to lower (5mg) doses.

Correlation Between the Use of Statins and Insulin Secretion and Sensitivity

Three studies assessed the link between statin use and insulin sensitivity [10,20,21]. All three studies found that statin use impairs insulin sensitivity. The association between statins and insulin insensitivity is dose-dependent, with higher doses associated with higher insulin insensitivity than lower doses [10,20].

Association of Statins Use and the Risk of Incident Type 2 Diabetes

Insulin resistance is a significant risk factor for type 2 diabetes, a metabolic condition defined by elevated blood glucose (sugar) levels [24]. Eight studies investigated the relationship between statins and the risk of incident type 2 diabetes [5,9,10,22-26]. Evidence from these studies shows that statin users are at higher risk of developing incident type 2 diabetes compared to non-statin users. The risk of developing incident type 2 diabetes ranges between 3.4% and 44% among statin users, while non-statin users’ range between 1.2% and 5.8% [5,9,10,22-26].

The association between statin usage and incident type 2 diabetes risk is dose-dependent, with higher doses (20mg) associated with a higher risk of incident type 2 diabetes compared to lower doses (5mg) [10,23,24].

Five studies also investigated the relationship between different types of statins and the risk of incidence of type 2 diabetes [9,10,22,24,25]. According to four of the five studies, the risk of developing incident type 2 diabetes is higher with potency statins such as atorvastatin, fluvastatin, pravastatin, rosuvastatin, lovastatin, and simvastatin compared to other statin types such as pitavastatin [10,22,24,25]. However, one study conducted by Ahmadizar et al. reported conflicting findings. According to this study, the increased risk of incident type 2 diabetes is independent of statin types [9].

Two studies investigated the duration of statin use and the risk of incident type 2 diabetes [9,23]. Evidence from the two studies shows that the risk of incident type 2 diabetes is time-dependent on the statin’s use; this implies that the likelihood of developing diabetes is notably greater in individuals who use statins for medium periods (31-365 days) or longer (>365 days) compared to those who use them for short periods (<31 days).

Study Strengths and Limitations

This research filled some gaps in the literature because no systematic reviews are looking into the relationship between statin therapy and a decline in insulin sensitivity/insulin resistance, which frequently results in the onset of diabetes. The findings of this investigation further support those of prior studies that found an association between statin medication and insulin intolerance [10,20,21].

While it is essential to acknowledge some limitations of the present study, it is also worth noting some of its strengths. First, the study was appropriately powered to identify meaningful correlations between insulin resistance and statin usage, provided they existed. Second, only top-notch studies were used to investigate the relationship between insulin intolerance and statin consumption. To the best of our knowledge, this is the most comprehensive and exhaustive systematic review of statin use and insulin resistance.

Despite these strengths of the study, it is crucial to interpret the findings in the context of the study's limitations: Because the research findings were qualitatively combined rather than quantitatively, it may be challenging to extrapolate their conclusions to a broader range of individuals. The analysis comprises several studies that failed to consider certain variables that could lead to a disparity in insulin resistance between the two groups, even with statin use. Factors such as race and ethnicity, family history of diabetes, cholesterol levels, body mass index, and the presence or absence of prediabetes were not accounted for in some studies. The absence of data from other large-scale trials reduced the statistical power of our study. The original authors were contacted for unpublished information, but no reply was received. As mentioned earlier, some studies reported contradictory findings.

Study Implications

Despite statins being widely recognized for their effectiveness in preventing cardiovascular disease in both primary and secondary cases, a growing body of research, including the present study, indicates that statins may lead to decreased insulin sensitivity, a significant risk factor for incident type 2 diabetes. Based on these previous discoveries, the FDA updated the labels for statins to include a cautionary note regarding the increased possibility of developing incident diabetes mellitus [29]. Statins have been found to decrease glycemic control, elevate fasting glucose levels, and raise insulin resistance, all of which could potentially contribute to the development of diabetes mellitus. However, the precise mechanism by which statins cause incident diabetes remains uncertain.

Carter et al. [22] and Olotu et al. [24] suggested there may be a need for doctors to individualize statin therapy, especially among people with low cardiovascular risk, because types and doses of statins differ in their capacity to reduce low-density lipoprotein cholesterol (i.e., the "bad" cholesterol) as well as in their potential to cause diabetes. The statins' type and dosage, the patient's overall cardiovascular risk and metabolic profile, and sound clinical judgment should all be the foundation of this customized therapy [22,24]. Pravastatin, which appears to be the least diabetogenic statin currently on the market, may be the best option for individuals with hyperlipidemia who have a high propensity for diabetes but a low risk of cardiovascular disease, according to Carter et al. [22]. This claim is also supported by the study's findings, which show that pravastatin and rosuvastatin users had the lowest levels of insulin resistance. However, it is essential to note that even though possibility that statin therapy may cause a reduction in insulin sensitivity, there is evidence that the cardiovascular advantages of statin therapy outweigh this possibility.

## Conclusions

In conclusion, the present systematic review provides compelling evidence that statins are significantly associated with a decrease in insulin sensitivity, regardless of the type of statin used. These findings have important clinical implications for healthcare providers in managing patients with high cholesterol. However, a targeted approach may optimize treatment outcomes by selecting statins with a lower risk of causing insulin intolerance in patients at a higher risk of developing diabetes. This approach can help healthcare providers personalize treatment plans to meet the individual needs of their patients and achieve better health outcomes.
